# Matrix Effect in the Isolation of Breast Cancer-Derived Nanovesicles by Immunomagnetic Separation and Electrochemical Immunosensing—A Comparative Study

**DOI:** 10.3390/s20040965

**Published:** 2020-02-11

**Authors:** Silio Lima Moura, Mercè Martì, María Isabel Pividori

**Affiliations:** 1 Grup de Sensors i Biosensors, Departament de Química, Universitat Autònoma de Barcelona, 08193 Bellaterra, Spain; siliosilicio@hotmail.com; 2 Laboratori d’Immunologia Cel·lular, Institut de Biotecnologia i Biomedicina, Universitat Autònoma de Barcelona, 08193 Bellaterra, Spain; merce.marti@uab.cat

**Keywords:** exosomes, breast cancer biomarker, liquid biopsy, immunomagnetic separation, ultracentrifugation, electrochemical immunosensor

## Abstract

Exosomes are cell-derived nanovesicles released into biological fluids, which are involved in cell-to-cell communication. The analysis of the content and the surface of the exosomes allow conclusions about the cells they are originating from and the underlying condition, pathology or disease. Therefore, the exosomes are currently considered good candidates as biomarkers to improve the current methods for clinical diagnosis, including cancer. However, due to their low concentration, conventional procedures for exosome detection including biosensing usually require relatively large sample volumes and involve preliminary purification and preconcentration steps by ultracentrifugation. In this paper, the immunomagnetic separation is presented as an alternative method for the specific isolation of exosomes in serum. To achieve that, a rational study of the surface proteins in exosomes, which can be recognized by magnetic particles, is presented. The characterization was performed in exosomes obtained from cell culture supernatants of MCF7, MDA-MB-231 and SKBR3 breast cancer cell lines, including TEM and nanoparticle tracking analysis (NTA). For the specific characterization by flow cytometry and confocal microscopy, different commercial antibodies against selected receptors were used, including the general tetraspanins CD9, CD63 and CD81, and cancer-related receptors (CD24, CD44, CD54, CD326 and CD340). The effect of the serum matrix on the immunomagnetic separation was then carefully evaluated by spiking the exosomes in depleted human serum. Based on this study, the exosomes were preconcentrated by immunomagnetic separation on antiCD81-modified magnetic particles in order to achieve further magnetic actuation on the surface of the electrode for the electrochemical readout. The performance of this approach is discussed and compared with classical characterization methods.

## 1. Introduction

Breast cancer is a highly lethal malignancy and the most commonly diagnosed cancer among women (WHO), with an estimated over 2 million new cases in 2018 [1]. Although treatments have increased the survival rate, it is still considered a major cause of morbidity and mortality in women. Treatment efficacy is highly related to early diagnosis. There is thus a growing demand for biomarkers that can help detect diseases at an early stage, as well as for follow-up of patients and therapeutic strategies. The exosomes [2] have received increasingly attention in the last years as a biomarker for early cancer detection and monitoring [3]. Exosomes are extracellular nanovesicles of 30–150 nm released by all types of cells during fusion of the multivesicular endosomes (MVEs) with the plasmatic membrane. They carry a cargo of active molecules to proximal and distal cells of the body as a mechanism of physiological communication for maintaining the natural homeostasis or even for a pathological response [4]. All types of cells use exosomes for this purpose. Importantly, one of the most remarkable features is that they are present in all the biological fluids, such as blood [5], saliva [6] and urine [7], among others. Their easy accessibility is one of the most compelling reasons for developing exosomes as clinical biomarkers. Another striking characteristic is their molecular cargo, which can be useful for diagnosis and prognosis of several diseases and conditions. During the biogenesis, some components of the cell of origin remain in the exosomes. Therefore, they carry specific surface markers that indicate their cell signatures, including surface proteins such as proteins related to transport and fusion (e.g., flotillin, caveolin-1), tetraspanins (e.g., CD9, CD63, CD81), heat shock proteins (e.g., Hsp90) and lipid-related proteins, as well as micro RNA (miRNA), messenger RNA (mRNA) and DNA [8]. However, realizing the potential of these vesicles as biomarker requires technical improvement, since the exosomes are exceptionally challenging to characterization with current technologies. The most common methods for targeting exosomes to date typically involve purification [9] followed by the specific characterization of their cargo [10]. The characterization firstly relies on morphological analysis ^10^. The nanometric size of exosomes puts them out of the sensitivity range of most cell-oriented sorting or analysis platforms, as is the case of the classical flow cytometers. Exosomes can only be visualized with an electron microscope. Nanoparticle tracking analysis (NTA) is usually used to count the exosomes, followed by downstream processes for specific detection, including LC-MS/MS, Western Blot, and RT-PCR.

Furthermore, due to their low concentration, conventional procedures for exosome characterization and detection usually require relatively large sample volumes and involve a preliminary purification and preconcentration step by ultracentrifugation, precipitation, size-exclusion chromatography or ultrafiltration, in order to prevent interferences from cells and free biomolecules in the sample. Differential centrifugation was the first technique described [2] and later optimized [10] by increasing *g* values (ultracentrifugation), being currently considered the gold standard for exosomes isolation. The main goal is to eliminate interferences of dead cells, cell debris and soluble protein by a physical separation. The major drawback is the lack of specificity, since it separates the whole population of exosomes, regardless their cell origin. Moreover, it can produce mechanical damage. The whole procedure is thus time consuming, requiring skilled personnel as well as laboratory facilities and benchtop instrumentation.

Therefore, there is a growing need for novel methods to accurately characterize and specifically isolate exosomes in complex biological fluids.

Since the early reports on magnetic separation technology [11], magnetic particles (MPs) have been used as a powerful and versatile preconcentration tool in a variety of analytical and biotechnology applications [12] and in emerging technologies including microfluidic devices and biosensors [13,14,15,16]. Magnetic particles (core such as iron, nickel, neodymium or magnetite) can be easily functionalized with biomolecules (for instance antibodies) to specifically bind the exosomes and concentrate them from the complex matrix under magnetic actuation, avoiding the interference of the biofluid matrix. Exosomes are thus preconcentrated while the interfering matrix is removed at the same time, increasing the sensitivity of the detection. Recent advances demonstrated the feasibility of the integration of MPs into bioassays for the detection and quantification of exosomes. We have recently demonstrated the feasibility of particle-based magnetic enrichment for exosomes isolation avoiding the ultracentrifugation steps in undiluted human serum using a magneto-actuated enzyme-linked immunosorbent assay (magneto-ELISA), which simplifies the conventional ELISA [17].

Furthermore, magnetic particles can be actuated on the surface of electrochemical transducers [13,16]. This kind of approach simplifies the analytical procedure in order to be used in resource-scarce settings by unskilled personnel, for the rapid detection of a condition, such as breast cancer, the most commonly diagnosed cancer among women [1]. Most of the currently available technologies for breast cancer diagnosis are based on imaging techniques [18]. The liquid biopsies based on circulating tumor cells (CTCs) can potentially simplify the equipment requirements and operational costs [19,20]. However, the clinical use of liquid biopsies is limited by their rarity in the peripheral blood [21] (1 CTC in 1 × 10^5–6^ blood cells), hindering their detection even with benchtop equipment and requiring preconcentration from a large volume of sample [22]. However, a single breast cancer cell can release up to 60–65 exosomes per hour [23].

In this paper, we compare the analytical performance of immunomagnetic separation for the isolation of nanovesicles from breast cancer cell lines spiked in serum, and the further detection by a magneto-actuated electrochemical immunosensor. To achieve that, the culture supernatant from three breast cancer cell lines, including MCF7 (ATCC^®^ HTB-22™), MDA-MB-231 (ATCC^®^ CRM-HTB-26™) and SKBr3 (ATCC^®^ HTB-30™) were purified by ultracentrifugation and spiked in phosphate buffer and depleted human serum at a wide concentration range, in order to carefully evaluate the effect of the human serum matrix and the presence of free receptors in the immunomagnetic separation (IMS). The results suggest that the use of CD81 modified magnetic particles can be useful for the specific isolation of exosomes, and further labeling using general as well as cancer-related receptors for the electrochemical readout.

## 2. Materials and Methods

### 2.1. Instrumentation

Nanoparticle tracking analysis (NTA) was performed using the NanoSight LM10-HS system with a tuned 405 nm laser (NanoSight Ltd., Salisbury, UK). The cryogenic transmission electron microscopy (TEM) images were collected by a Jeol JEM 2011 (JEOL USA Inc, Peabody, MA, USA) transmission electron microscope at an accelerating voltage of 200 kV. The confocal images were collected on the microscope Leica, TCS SP5 (Leica Microsystems, Wetzlar, Germany). Flow cytometry was performed using BD FACSCANTO II (BD Biosciences, San Jose, CA, USA) equipment. The media fluorescence intensity (MFI) and beads count data of every sample-reading file were obtained by FlowJo analysis software. Optical measurements were performed on a TECAN Sunrise (TECAN AG, Männedorf, Switzerland) microplate reader with Magellan v4.0 software. Polystyrene microtiter plates were purchased from Nunc (Maxisorp, Roskilde, Denmark). All electrochemical experiments were performed using an AUTOLAB PGSTAT10 (Metrohm, Herisau, Switzerland) potentiostat/galvanostat electrochemical analyzer. The data were in all cases fitted using a nonlinear regression (four parameters logistic equation).

### 2.2. Chemicals and Biochemicals

Magnetic particles (Dynabeads^®^ M450 Tosylactivated, nº 14013, 4.5 µm diameter) were purchased from Thermo Fisher (Waltham, MA, USA). The antibodies are summarized in Table 1. The antibodies were selected towards different biomarkers that were previously reported to be expressed in the surface of exosomes, as shown in Table 2. The 10 mmol L^−1^ phosphate-buffered saline (PBS) buffer solution (pH 7.5) and boric acid buffer solution (pH 8.5) were prepared with ultrapure water and all other reagents were in analytical reagent grade (supplied from Sigma Aldrich, St. Louis, MO, USA). The Pierce™ TMB Substrate Kit (Ref. 34021) was purchased from Thermo Fisher. The Hoechst dye (No. 62249, Thermo Fisher) and Cy^®^5 fluorophore dye (anti-mouse, No. ab97037, Abcam, Cambridge, UK) used in confocal microscopy were purchased from distributors in Barcelona, Spain. Ultrapure water (Millipore^®^ System, resistivity 18.2 MΩ cm) was used throughout the experiments.

### 2.3. Cell Culture

The breast cancer cell lines were the following: MCF7 (ATCC^®^ HTB-22™), MDA-MB-231 (ATCC^®^ CRM-HTB-26™) and SKBr3 (ATCC^®^ HTB-30™). Expansion of cell population was carried out from 1,000,000 cells in a T-175 flask containing 32 mL of Dulbecco’s Modified Eagle Medium (DMEM) (Ref. 31966047, Thermo Fisher), supplemented with 10% exosome-depleted fetal bovine serum (FBS) (Ref. 12007C, Sigma-Aldrich, St. Louis, MO, USA), 100 U mL^−1^ penicillin-streptomycin (Ref. 15140122, Thermo Fisher). The temperature was maintained at 37 °C in a humidified, concentrated CO_2_ (5%) atmosphere. Once cells reached approximately 95% confluence on the T-175 flask, the culture medium was removed and immediately centrifuged (300 g for 10 min, 2000 g for 10 min and 10000 g for 30 min) and stored at −80 °C until exosome isolation was achieved.

### 2.4. Exosome Isolation and Purification

**Figure 1:**
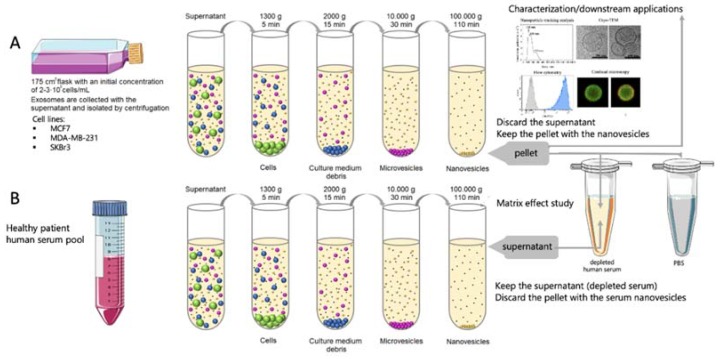
(**A**) Schematic procedure for the isolation of exosomes from cell culture supernatant of breast cancer cells. (**B**) Schematic procedure for the obtaining of depleted human serum. The undiluted serum (as well as 10%, 25% and 50% dilutions in phosphate-buffered saline (PBS)) is then spiked with the purified exosomes, as well as PBS as a control, in order to perform the matrix effect study.

Exosomes were purified according to Théry [10] and as schematically shown in Figure 1, panel A. The supernatant from MCF7, MDA-MB-231 and SKBr3 breast cancer cell lines were subjected to differential centrifugation as follows: 1300 g for 5 min (removal of residual cells), 2000 g for 15 min and 10,000 g for 30 min (removal of cellular debris). Subsequently, a Beckman Coulter Optima^TM^ L-80XP Ultracentrifuge was used at 100,000 g for 60 min with a 70Ti rotor to pellet exosomes. After that, the supernatant was carefully removed, and crude exosome-containing pellets were resuspended in 10 mmol L^−1^ phosphate-buffered saline (PBS) solution and pooled. A second round of the same ultracentrifugation setting was carried out, and the resulting exosome pellet was resuspended in 500 µL (per 100 mL of supernatant) of 10 mmol L^−1^ PBS buffer solution (0.22 µm filtrated and sterile) and stored at −80 °C. All centrifugation steps were performed at a temperature of 4 °C.

### 2.5. Exosome-Depleted Human Serum

The exosome-depleted human serum was used for several matrix effect studies. The depletion of exosomes in human serum from healthy patients was performed as described above, and is schematically shown in Figure 1, panel B. This exosome-depleted human serum was spiked at a different concentration range with the exosomes obtained as above (Figure 1, panel A).

### 2.6. Covalent Immobilization on Magnetic Particles

Dynabeads^®^ M450 tosylactivated superparamagnetic particles (MPs, 4.5 µm in diameter) have a core of iron oxide salt encapsulated by a polystyrene polymer, which has a polyurethane external layer with the p-toluenesulfonate group [71]. It is a good leaving group, which allows an S_N_2 reaction to occur in the presence of a nucleophile [72]. A nucleophilic reaction by an antibody, protein, peptide or glycoprotein removes and replaces the sulfonyl ester groups from the polyurethane layer.

**Figure 2:**
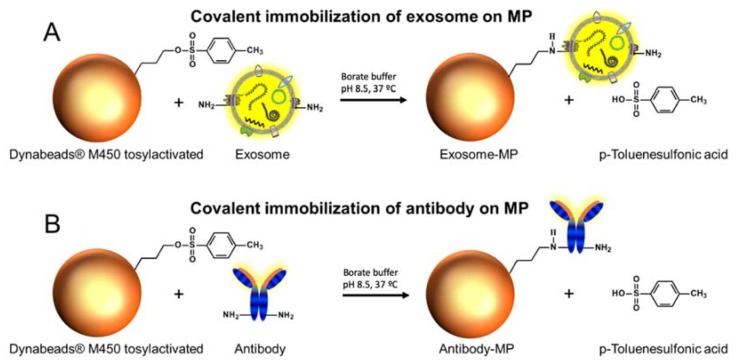
(**A**) Direct covalent immobilization on Dynabeads^®^ M450 tosylactivated. (**B**) Covalent immobilization of different antibodies (as depicted in Table 1) on Dynabeads^®^ M450 tosylactivated.

Two different approaches were used, as depicted in Figure 2. The first one involves the direct covalent immobilization of exosomes on magnetic particles (Figure 2, panel A). The second approach is based on the covalent immobilization of the antibodies for a further immunomagnetic separation (IMS) of exosomes (Figure 2, panel B).

*Immobilization of exosomes.* The immobilization of exosomes on Dynabeads^®^ M450 tosylactivated superparamagnetic particles (MPs) (Figure 2, panel A) was performed as follows: 3.5 × 10^10^ exosomes were added to 40 µL (1.6 × 10^7^ MPs) tosylactivated Dynabeads^®^ M450. The reaction kinetics were increased by adding a 0.1 mol L^−1^ pH 8.5 borate buffer, in order to ensure the nucleophilic reaction by the amine group. The incubation step was performed overnight with gentle shaking at 4 °C. After that, a 0.5 mol L^−1^ glycine solution was added to ensure the blocking of any remaining tosylactivated groups, by incubation for 2 h at 25 °C. After that, the exosome-modified magnetic particles (exosomes-MP) were resuspended in 160 µL of 10 mmol L^−1^ PBS buffer solution in order to achieve 1 × 10^6^ MPs per 10 µL. The calibration plots were performed by using 1 × 10^6^ MPs per well plate and the number of exosomes ranged from 3.9 × 10^4^ to 2 × 10^7^ exosomes/µL^−1^.

*Immobilization of antibodies.* The different specific antibody (approximately 15 μg mL^−1^ antiCDX, optimized in each case as described in detail by our research group [17] to achieve full coverage) was added to 55 µL (2.2 × 10^7^ MPs) Dynabeads^®^ M450 tosylactivated (Figure 2, panel B). As above, 0.1 mol L^−1^ pH 8.5 borate buffer was added. The incubation step was performed overnight with gentle shaking at 37 °C. After that, a blocking step with 0.5 mol L^−1^ glycine solution was performed for 2 h to ensure the blocking of the any remaining tosylactivated groups. After that, the antibody-modified MPs (herein, antiCDX-MPs, where antiCDX = antiCD9, antiCD24, antiCD44, antiCD54, antiCD63, antiCD81, antiCD326 or antiCD340) were resuspended in a 220 µL (10 µL per well in order to achieve 1 × 10^6^ particles per well) 10 mmol L^−1^ PBS buffer. It was not possible to immobilize CD326 satisfactorily on MPs.

### 2.7. Characterization of Exosomes by Nanoparticle Tracking Analysis

Nanoparticle tracking analysis (NTA) was used as a gold standard method to count the exosomes. This information was used for the biosensing calibration plots [73,74]. NTA was also used to study the size distribution of exosomes purified from cell culture supernatant. The size distribution and concentration of exosomes were measured using the NanoSight LM10-HS system with a tuned 405 nm laser (NanoSight Ltd., Amesbury, UK). The purified exosomes were diluted in sterile-filtered PBS (50- to 100-fold). Nanosight NTA Software analyzed raw data videos by triplicate during 60 s with 50 frames per second and the temperature of the laser unit set at 24.8 °C.

### 2.8. Characterization of Exosomes by Transmission Electron Microscopy

A 10 µL aliquot of exosomes was directly laid on Formvar–carbon electron microscopy grids and frozen in ethanol. The Cryo-TEM images were collected on the on a Jeol JEM 2011 transmission electron microscope at an accelerating voltage of 200 kV. Exosomes were maintained at −182 °C during the whole process.

### 2.9. Confocal Microscopy Study

Confocal microscopy was used for the assessment of the molecular biomarkers expressed in the exosomes obtained by the three different cancer cell lines (MCF7, MDA-MB-231 and SKBr3). The presence of the following receptors was investigated: CD9, CD63, CD81, CD24, CD44, CD54, CD326 and CD340. As exosomes are between 30–200 nm in diameter, a size that makes them out of the sensitivity range of most cell-oriented sorting or analysis platforms, they were coupled to MPs to allow their characterization by confocal microscopy. In this study, the exosomes were attached on the surface of MPs by covalent immobilization (exosomes-MPs), as described in Figure 1, panel A. In order to achieve that, 3.5 × 10^10^ exosomes were covalently immobilized on 1.6 × 10^7^ MPs overnight with gentle orbital shaking at 4 °C. The indirect labeling was performed by incubation with 100 µL (5 µg mL^−1^) of the mouse antibodies towards the different cluster of differentiation antiCDX (CDX being either CD9, CD63, CD81, CD24, CD44, CD54, CD326 or CD340 biomarkers), for 30 min with gentle shaking at 25 °C. Subsequently, three washing steps were performed. Afterwards, 100 µL (2 µg mL^−1^) of antimouse-Cy5 antibody (a far-red-fluorescent dye, excitation 647 nm, emission 665 nm [75]) was incubated for 30 min in darkness, with gentle shaking at 25 °C, for further readout. In all instances, the percentage of labeled entities was normalized by the highest fluorescence value for a labeled receptor. The labeled exosomes-MP were resuspended in 200 μL of PBS with 0.1% bovine serum albumin (BSA) solution. After each incubation, washing steps with PBS with 0.1% BSA solution were performed. The confocal images were then collected.

### 2.10. Flow Cytometry Study

The analysis of the molecular biomarkers CD9, CD24, CD44, CD54, CD63, CD81, CD326 and CD340 expressed in the exosomes derived from three different cancer cell lines (MCF7, MDA-MB-231 and SKBr3) was performed by flow cytometry. Accordingly, for the labeling of cell-derived exosomes, they were firstly immobilized on the surface of MPs by two different approaches, as described above (Figure 2). In Figure 2, panel A depicts the covalent immobilization of the 3.5 × 10^10^ exosomes on 1.6 × 10^7^ MPs (exosomes-MPs). The exosomes-MPs were analyzed by indirect labeling by incubation with 100 µL (5 µg mL^−1^) of the antibodies antiCDX (mouse), (CDX being either CD9, CD24, CD44, CD54, CD63, CD81, CD326 or CD340 biomarkers), for 30 min with gentle shaking at 25 °C. After that, three washing steps with PBS pH 7.5 0.5% BSA were performed. Afterwards, 100 µL (2 µg mL^−1^) of antimouse-Cy5 antibody (a far-red-fluorescent dye, excitation 647 nm, emission 665 nm) was incubated for 30 min at 25 °C. The labeled exosomes-MPs were resuspended in PBS pH 7.5 0.5% BSA. In Figure 1, panel B, 1 × 10^6^ MPs modified with rabbit antiCD81 (antiCD81-MPs) were incubated with 4 × 10^9^ exosomes (1:4000 MP/exosomes ratio). Exosomes immunocaptured by antiCD81-MPs were detected using indirect labeling, as described above.

### 2.11. Matrix Effect Study by Immunomagnetic Separation and Optical Readout

The evaluation of the matrix effect was firstly performed by immunomagnetic separation (IMS) of MCF7-derived exosomes spiked in 0% (10 mmol L^−1^ phosphate-buffered saline (PBS) (no serum)), 10%, 25%, 50% and 100% (undiluted) exosome-depleted human serum (obtained as schematically shown in Figure 1, panel B). The IMS was performed by antiCDX-MPs (CDX being either CD9, CD24, CD44, CD54, CD63, CD81, CD326 or CD340 biomarkers), followed by a direct labeling and optical readout. The procedure for the magneto-actuated immunoassay was previously described in detail by our research group [17]. To summarize, this approach involved a direct immunoassay format, and in all cases was performed in 96-well microtiter plates. The direct immunoassay format involved the following steps: (i) IMS of the exosomes with antiCDX-MPs. The antiCDX-MPs (CDX being any of CD9, CD24, CD44, CD54, CD63, CD81, CD326 or CD340 biomarkers) (containing 1 × 10^6^ antiCDX-MPs per well) and the exosomes (4 × 10^9^ exosomes per well spiked in 100 μL of any of the matrix: 0%, 10%, 25% or 50% diluted and undiluted human serum), were simultaneously incubated for 30 min with shaking at 25 °C, followed by three washing steps with PBS containing 0.5% BSA. (ii) Direct labeling. The exosomes-coated MPs were incubated with the antiCD63-HRP antibody (100 μL, 1.24 µg mL^−1^) for 30 min with shaking at 25 °C, followed by three washing steps with PBS containing 0.5% BSA. (iii) Optical readout. 100 μL of substrate solution (0.004% *v/v* H_2_O_2_ and 0.01% *w/v* TMB in citrate buffer) was then added to each well and incubated for 30 min under dark conditions. The enzymatic reaction was stopped by adding 100 μL of H_2_SO_4_ (2.0 mol L^−1^). Then, exosomes-MPs were separated using a magnet plate separator, and an exosomes-MPs pellet formed on the bottom tube, followed by supernatant separation. The absorbance measurement of the supernatants was thus performed with the microplate reader at 450 nm. After each incubation or washing step, a 96-well magnet plate separator was positioned under the microtiter plate until the pellet formation on the bottom corner, followed by supernatant separation.

### 2.12. Immunomagnetic Separation and Electrochemical Immunosensing of Exosomes in Human Serum

Two different formats, classified accordingly to the labeling, were performed for the detection of exosomes derived in this instance from SKBr3 breast cancer cell lines, and spiked in both phosphate buffer and undiluted exosome-depleted human serum, and ranging from 0 to 2 × 10^9^ exosomes, as determined by NTA. The whole procedure was performed in detail as follows:

*Direct format*. The magneto-actuated electrochemical immunosensing involves the following steps: (i) IMS of the exosomes with antiCD81-MPs (rabbit) (containing 1 × 10^6^ antiCD81-MPs per well) and the exosomes (100 μL, ranging from 0 to 2 × 10^9^ exosomes per well), were simultaneously incubated for 30 min with shaking at 25 °C, followed by three washing steps with PBS containing 0.5% BSA. (ii) Direct labeling. The modified-MPs were incubated with the antiCD63-HRP antibody (100 μL, 1.24 µg mL^−1^) for 30 min with shaking at 25 °C, followed by three washing steps with PBS containing 0.5% BSA. (iii) Electrochemical readout, as described below.

*Indirect format.* The magneto-actuated electrochemical immunosensing involves the following steps: (i) IMS of the exosomes with antiCD81-MPs (rabbit) (containing 1 × 10^6^ antiCD81-MPs per well) and the exosomes (100 μL, ranging from 0 to 2 × 10^9^ exosomes per well), were simultaneously incubated for 30 min with shaking at 25 °C, followed by three washing steps with PBS containing 0.5% BSA. (ii) Incubation with the antiCDX mouse monoclonal antibodies (100 μL, 0.50 µg mL^−1^) (CDX being any of the CD24 or CD340 biomarkers), which were simultaneously incubated for 30 min with shaking at 25 °C, followed by three washing steps with PBS containing 0.5% BSA. (iii) Indirect labeling. The modified-MPs were incubated with antimouse-HRP antibody (100 μL, 0.08 ng mL^−1^) for 30 min with shaking at 25 °C, followed by three washing steps with PBS containing 0.5% BSA. (iv) Electrochemical readout, performed as described below, after the magnetic actuation of the modified-MPs on the surface of the m-GEC electrodes.

**Figure 3:**
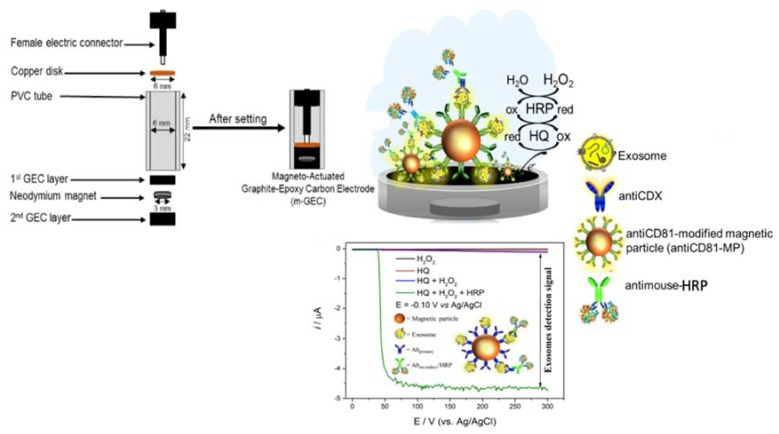
Schematic representation of the construction of the magneto-actuated electrode based on rigid composites (m-GEC) as well as the enzymatic reaction based on hydroquinone mediator.

*Electrochemical measurements.* All electrochemical experiments were performed using an AUTOLAB PGSTAT10 potentiostat/galvanostat electrochemical analyzer. A magneto-actuated graphite-epoxy carbon (m-GEC) as working electrode (geometric area = 0.5 cm^2^), a Ag/AgCl/KCl_(satd.)_ reference electrode, a platinum counter electrode (geometric area = 3.0 cm^2^) and a standard one-compartment three-electrode cell were used in all experiments. The enzymatic electrochemical signal is based on a hydroquinone mediator. Amperometric measurements were carried out at −0.1 V vs. Ag/AgCl/KCl_(satd.)_. All experiments were performed using a single m-GEC electrode in a 100 mmol L^−1^ phosphate-buffered saline (PBS) solution with pH 7.0 at 25 °C. A reproducible steady-current was obtained after 60 s and used for the calibration curve. An outline of this approach based on the magneto-actuated composite electrode and electrochemical readout is shown in Figure 3.

### 2.13. Safety Considerations

All procedures involved in the manipulation of human cells were handled using Biosafety Level 2 Laboratory (BSL-2) and containment. All works were performed in a Biosafety cabinet, and all materials were decontaminated by autoclaving or disinfected before discarding in accordance with the U.S. Department of Health and Human Services guidelines for level 2 laboratory Biosafety [76].

## 3. Results and Discussion

### 3.1. Characterization of Exosomes by Nanoparticle Tracking Analysis

**Figure 4:**
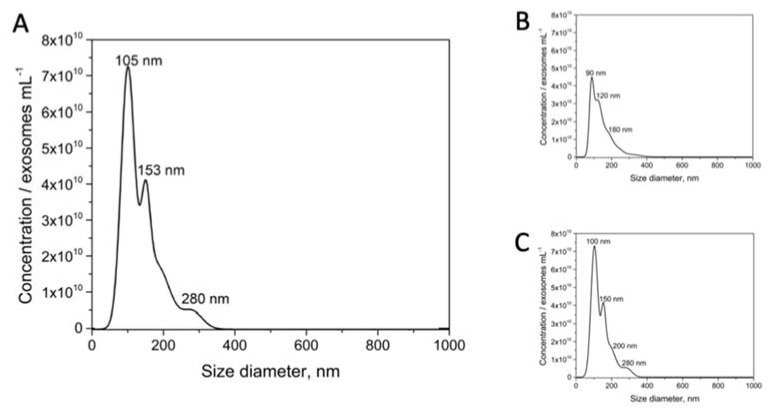
Nanoparticle tracking analysis (NTA) on size distribution on purified exosomes derived from (**A**) MCF7, (**B**) MDA-MB-231 and (**C**) SKBr3 breast cancer cell lines. The purified exosomes were diluted in sterile-filtered 10 mmol L^−1^ PBS buffer (pH 7.5). Nanosight NTA Software analyzed raw data videos by triplicate during 60 s with 50 frames per second and the temperature of the laser unit set at 24.8 °C.

An estimation of the size diameter distribution of purified exosomes derived from the MCF-7 breast cancer cell line was performed by NTA (Figure 4). A size from 50 to 300 nm (considering 95.4% of a Gaussian distribution), but with dominance around 105 and 153 nm (60% and 34% of the counted particles, respectively) was obtained for the MCF-7 exosomes. However, it is important to highlight that NTA analysis is not able to distinguish isolated particles from aggregates. Similar results were obtained by NTA for the exosomes derived from the other breast cancer cell lines (MDA-MB-231 and SKBr3) (as depicted in Figure 4, panels B and C, respectively).

### 3.2. Characterization of Exosomes by Transmission Electron Microscopy

**Figure 5:**
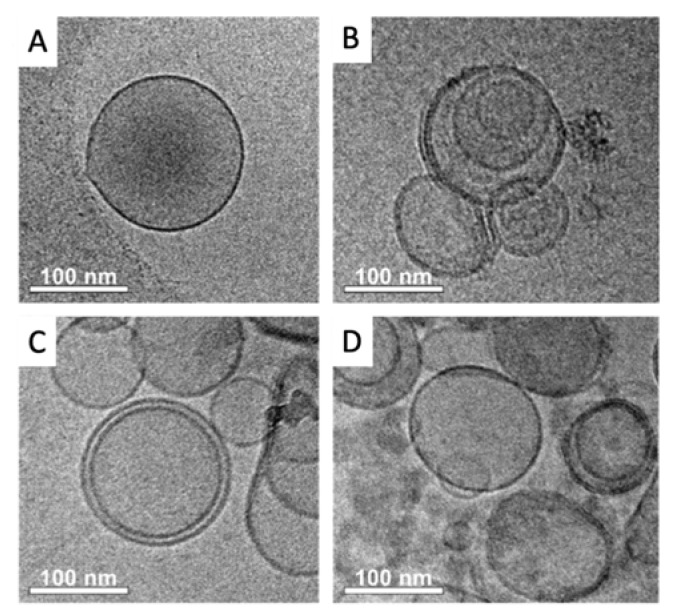
Cryogenic transmission electron microscopy (Cryo-TEM) on purified exosomes derived from (**A**, **B**) MCF-7, (**C**) MDA-MB-231 and (**D**) SKBr3 breast cancer cell lines.

Cryogenic transmission electron microscopy (TEM) provides a tool for high-resolution structural analysis for frozen biostructures, avoiding depression and even damage to the sample. Cryo-TEM micrographs on exosomes derived from breast cancer cell lines are shown in Figure 5.

The image comparatively shows TEM images of exosomes derived from MCF-7 (panels A and B), MDA-MB-231 (panel C) and SKBr3 (panel D) breast cancer cell lines. The micrographs revealed the typical exosome consisting of well-shape exosome vesicles with closed circular lipid bilayers comprising packed membrane proteins with a 110 nm diameter (Figure 5, panel A). As expected, the TEM micrographs also reveal the presence of some aggregates of exosomes (Figure 5, panel B), confirming that the NTA analysis cannot distinguish vesicles and vesicle aggregates. Similar results were obtained for exosomes derived from other breast cancer cell lines (MDA-MB-231 and SKBr3, Figure 5, panels C and D, respectively).

### 3.3. Confocal Microscopy Study

**Figure 6:**
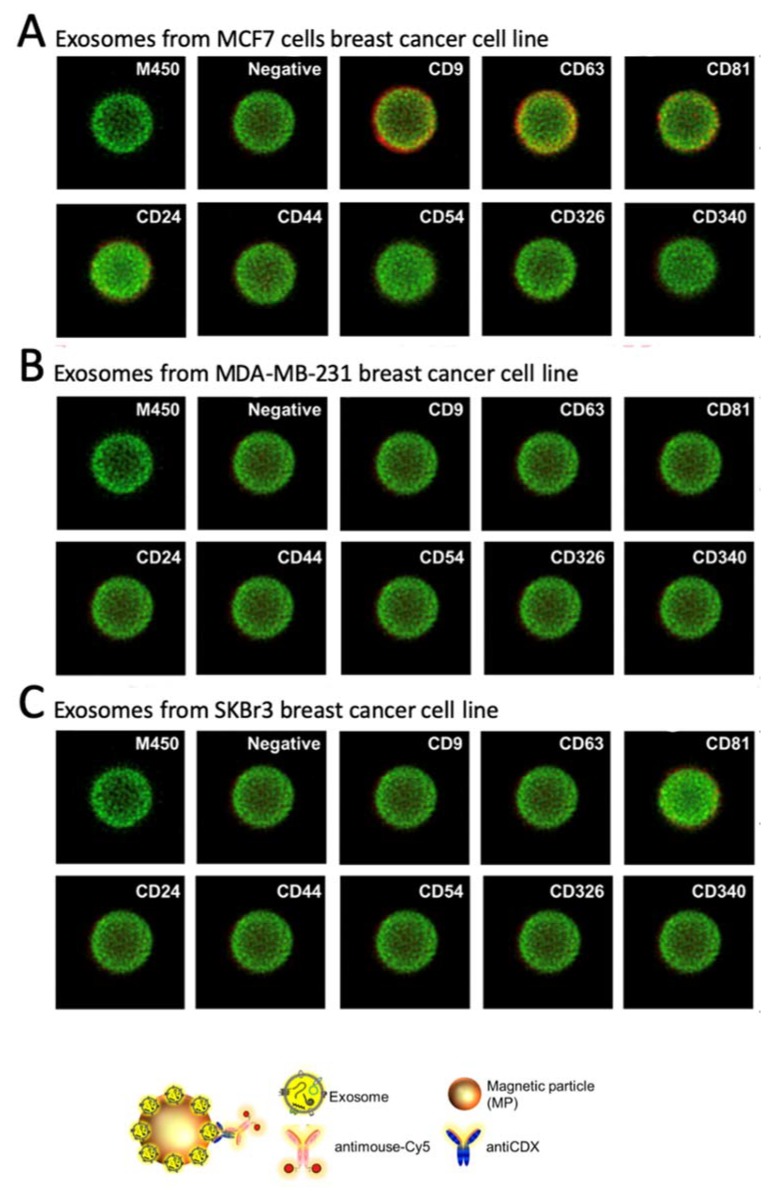
Confocal microscopy to evaluate the relative expression of CD9, CD63, CD81, CD24, CD44, CD54, CD326 and CD340 membrane protein markers in the exosomes derived from MCF7, MDA-MB-231 and SKBr3 breast cancer cell lines. Magnetic particles appear stained in green while the membrane protein receptors, in red, represents a positive expression on the membrane of the exosomes. In all cases, the primary antibody was 5 μg mL^−1^ and 2 µg mL^−1^ of antimouse-Cy5 antibody.

Confocal microscopy was performed to comparatively study the expression patterns of different biomarkers on breast cancer cells’ exosomes derived from MCF-7, MDA-MB-231 and SKBr3 cell lines. The quantitative results are summarized in Figure 6.

The expression of each cell line-derived exosomes was studied after the covalent immobilization on MPs (Figure 2, exosomes-MPs and Figure 6, panel A for exosomes derived from MCF7, panel B for MDA-MB-231 and panel C for SKBr3 cells lines). The intense green color of the magnetic particles is due to autofluorescence around 580 nm [77]. As expected, general tetraspanins (CD9, CD63 and CD81) [78,79] were expressed in exosomes from the MCF7 breast cancer cell line (Figure 6, panel A). The biomarkers related to breast cancer are weekly or not expressed. Negligible non-specific adsorption fluorescence was observed (Figure 6, negative control), indicating a good blocking procedure of the remaining tosyl-activated group after covalent immobilization.

On the other hand, a poorer labeling pattern was achieved for other biomarkers in MCF7 (Figure 4, panel A), as well those derived from MDA-MB-231 and SKBr3 (Figure 6, panels B and C). This issue can be attributed to steric hindrance of the receptor after immobilization of the exosomes on the MPs, in agreement with the results achieved by flow cytometry as it will be further discussed. It is worth mentioning that the confocal images are the most representative and they only provide a qualitative analysis.

### 3.4. Flow Cytometry Study

**Figure 7:**
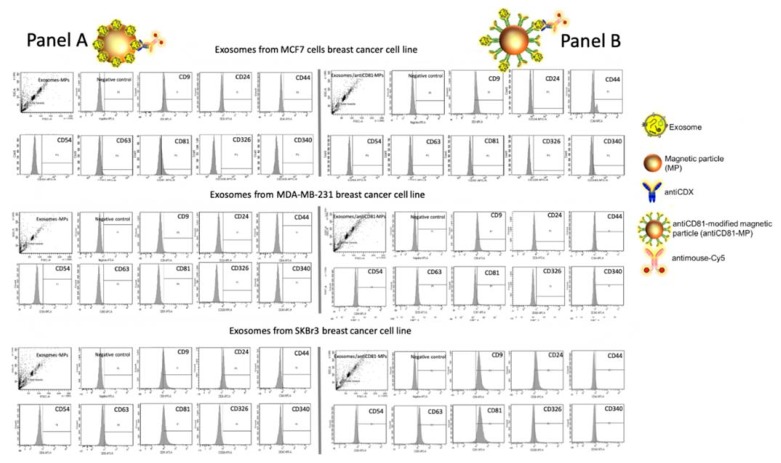
Flow cytometry study for exosomes derived from MCF7, MDA-MB-231 and SKBr3 breast cancer cell lines attached on magnetic particles (MPs), being (Panel A) covalent immobilization of the exosomes on MPs (exosomes-MPs) and (Panel B) IMS of the exosomes on antiCD81-MPs, followed by indirect labeling with mouse antiCDX (5 µg mL^−1^), (CDX being either CD9, CD24, CD44, CD54, CD63, CD81, CD326 or CD340 biomarkers) and antimouse-Cy5 (2 µg mL^−1^). The concentrations of MPs and exosomes were set at 1 × 10^6^ MPs and 4 × 10^9^ exosomes per assay, respectively.

As in the case of confocal microscopy, the main goal of this set of experiments was to assess the expression of different biomarkers (including general exosome biomarkers such as CD9, CD63 and CD81, and cancer-related biomarkers such as CD24, CD44, CD54, CD326 and CD340) on exosomes derived from the three different breast cancer cell lines (MCF7, MDA-MB-231 and SKBr3), in order to determine the good design for the electrochemical immunosensor. The results of flow citometry are shown in Figure 7, panels A and B. In the first approach, the exosomes directly immobilized on MPs were assessed towards the expression of different biomarkers selected in this study, followed by an incubation with an antimouse-Cy5 secondary antibody. Then, the labeled exosomes-MPs were acquired by the flow cytometer and further analyzed. As shown in Figure 7, panel A, CD9, CD63 and CD81 were expressed on exosomes produced by MCF7 and SKBr3, but poorly expressed in the MDA-MB-231 cell line, in agreement with confocal microscopy and several other studies [78,79]. Besides, cancer-related biomarkers showed, in general, a poorer labeling pattern. In the second approach, the two biomarkers must be simultaneously expressed in the exosomes. One of the markers is involved in the IMS, while the other one is involved in the labeling. Accordingly, the general tetraspanin CD81 was used in this instance for the IMS by the antiCD81-MPs in order to achieve a massive capture of the exosomes, followed by further labeling by a second biomarker. As shown in Figure 7, panel B, a better performance was achieved in general with this approach. Increased tetraspanin labeling on the exosomes from SKBr3 and MCF7 was respectively observed, although MDA-MB-231 also expressed low levels of these general biomarkers. Cancer-related exosomal biomarkers (for instance CD44 and CD340), which were negative when immobilized covalently on MPs, were detected by this approach. This experiment suggests that CD81 tetraspanin can be successfully used for the IMS of the exosomes. Moreover, and as expected, Figure 7 shows that there is a different pattern for the expression of exosomes from different cell cancer lines.

### 3.5. Matrix Effect Study by Immunomagnetic Separation and Optical Readout

**Figure 8:**
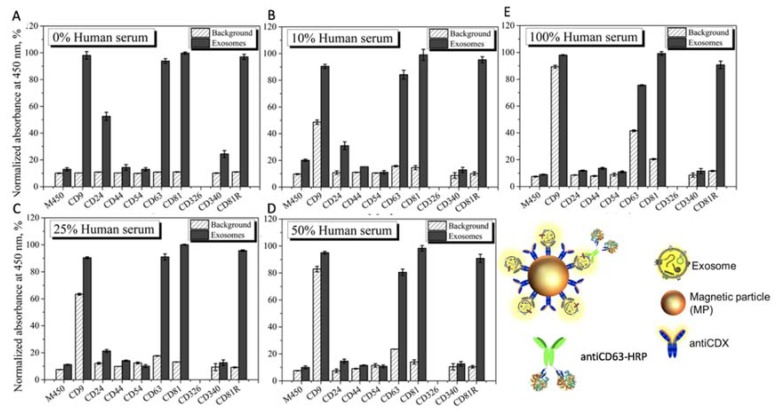
Evaluation of the matrix effect in exosome-depleted human serum for MCF7 exosomes detection using antiCDX-MPs modified with antibodies against CD9, CD24, CD44, CD54, CD63, CD81, CD340 and CD81R (rabbit polyclonal) biomarkers for the IMS, followed by direct labeling with an antiCD63-HRP antibody (1.24 μg mL^−1^). Detection of exosomes in (**A**) 0%; 10 mmol L^−1^ phosphate-buffered saline/PBS (standard for no matrix effect), (**B**) 10%, (**C**) 25%, (**D**) 50% and (**E**) 100% exosome-depleted human serum (undiluted human serum). The concentrations of MPs and exosomes were set at 1 × 10^6^ MPs and 4 × 10^9^ exosomes per assay, respectively. The background (as a negative control without exosomes) are also shown in all the experiments. The error bars show the standard deviation for n = 3.

The main goal of this study is to avoid the use of ultracentrifugation for the isolation of exosomes from the free proteins of the sample, and to replace it by immunomagnetic separation directly performed in undiluted human serum, in order to simplify the analytical procedure. This is a challenging task, since the free receptors (mainly proteins including tetraspanin) present in the undiluted serum can block—or even prevent—the separation of the exosomes by immunomagnetic separation. Accordingly, a rational study of the matrix effect of the human serum was performed, by spiking a known amount of MCF7 cancer-related exosomes (obtained as schematically shown in Figure 1, panel A), in undiluted human serum depleted of exosomes (obtained as schematically shown in Figure 1, panel B). The characterization of the interference of free molecules and proteins in the serum was then carefully evaluated by magneto-actuated immunoassay (optical readout), since it can be easily multiplexed. A preliminary study of the matrix effect of human serum was performed on exosomes purified by ultracentrifugation from the MCF7 cell line (as depicted in Figure 1, panel A) and spiked in 0% (PBS), 10%, 25%, 50% and 100% (undiluted) human serum. The signal was normalized by the highest absorbance value for a biomarker within each exosome immobilization approach, as shown in Figure 8.

**Figure 9:**
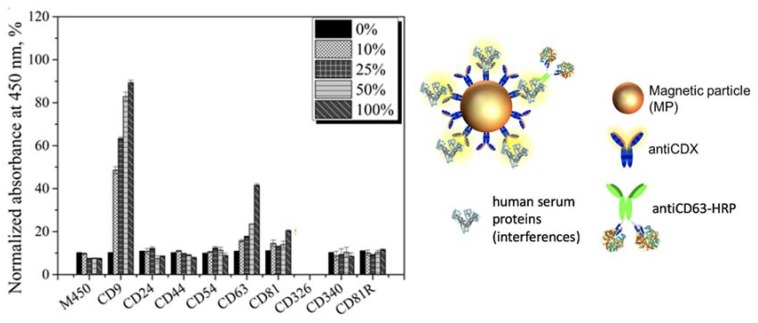
Evaluation of the matrix effect in exosome-depleted human serum using antiCDX-MPs modified with antibodies against CD9, CD24, CD44, CD54, CD63, CD81, CD340 and CD81R (rabbit polyclonal) biomarkers for the IMS, followed by direct labeling with an antiCD63-HRP antibody (1.24 μg mL^−1^) in depleted human serum. In this instance, no exosomes were spiked in the samples. All other conditions are as in Figure 8. The error bars show the standard deviation for n = 3.

In this approach, the IMS was performed by using a specific biomarker antibody (antiCDX-MPs), followed by an optical readout using a general exosome biomarker (antiCD63-HRP antibody). This format thus requires the coexistence of two biomarkers in the same exosomes to be magneto-actuated and labeled. By comparing the pattern of signal for each of the receptors used to separated the exosomes, the better analytical performance was achieved by using the ubiquitous and highly expressed CD81 for the immunomagnetic separation with antiCD81-MPs, since no background was observed and the value was always negligible at all the matrix compositions studied. On the contrary, a high background was observed in the case of using antiCD9-MPs. This high value can be attributed to a cross reaction/interference of some component of the serum with the antibodies used in this approach. The results also show the interference of the free CD63 protein in the serum, which produces increasing backgrounds as the percentage of human serum increases in the matrix. This free CD63 receptor can thus block, and therefore prevent by competition, the binding of the exosomes to the magnetic particles. These results were confirmed by performing the same experiments in depleted human serum (no exosomes present), and the results are shown in Figure 9.

Once again, it is demonstrated that some soluble proteins in the serum produce an increment in the signal, especially remarkable in the case of CD9 and CD63. Again, the CD81 commercial antibody (rabbit) is promising for the IMS of exosomes since no background is observed.

These results are also in agreement with previous studies, reporting a higher biomarker expression of CD9, CD63 and CD81 tetraspanins as well as a lower biomarker expression of CD24, CD44, CD54, CD326 and CD340 cancer-related biomarkers on breast cancer exosomes. No results were obtained for CD340 (Epcam), since upon immobilization on the MP, no binding was observed, perhaps due to a bad orientation during covalent immobilization.

### 3.6. Immunomagnetic Separation and Electrochemical Immunosensing of Exosome in Human Serum

**Figure 10:**
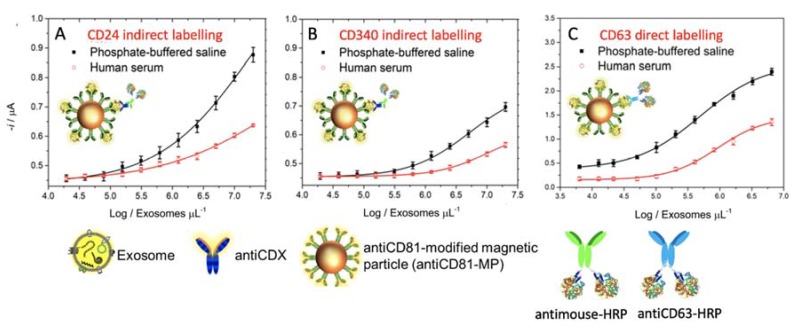
Electrochemical immunosensor for the detection of exosomes derived from the SKBr3 breast cancer cell line spiked in exosome-depleted human serum and a phosphate buffer. IMS of the exosomes on antiCD81-MPs, followed by indirect labeling with (**A**) antiCD24, (**B**) antiCD340 and by direct labeling with (**C**) antiCD63-HRP antibody. In all cases, the concentration of MPs was fixed in 1 × 10^6^ MPs, 0.50 μg mL^−1^ of primary antibody, 0.08 ng mL^−1^ of antimouse-HRP and 1.24 µg mL^−1^ of antiCD63-HRP antibody. Enzymatic electrochemical signals were monitored at −0.1 V vs. Ag/AgCl_(sat.)_. In all cases, the replicates were obtained with three different samples measured with different m-GEC electrodes. The error bars show the standard deviation for n = 3.

The detection of exosomes in non-diluted human serum is critical for a routine clinical diagnosis, since it can suffer from interferences of free biomarkers. In order to avoid ultracentrifugation, the evaluation of the analytical performance of the electrochemical immunosensor coupled with IMS based on a CD81 receptor for the detection of SKBr3 exosomes spiked in depleted non-diluted human serum is shown in Figure 10. The exosomes concentration ranged from 2.00 × 10^7^ to 2.00 × 10^10^ exosomes/mL^−1^ in order to cover the matrix effect in a wide concentration range. The exosomes concentration in healthy individuals determined by NTA in biological fluids varies notably in the literature, from 1.505 to 2.245 × 10^8^ vesicles/mL^−1^ (plasma of healthy male individuals) [80]; 9.25 × 10^9^/2.4 × 10^10^/1.8 × 10^10^ vesicles/mL^−1^ (depending on the mode of NTA in plasma of healthy individuals) [81]; 0.7 × 10^8^/5.3 × 10^8^ vesicles/mL^−1^ (serum)/2.3 × 10^8^/9.9 × 10^8^ vesicles/mL^−1^ (plasma) (depending on the mode of NTA in plasma of healthy individuals) [82]; and 0.88 × 10^8^ to 1.34 × 10^9^ exosomes/mL^−1^ (serum or plasma of healthy individuals) [83].

The results show the performance of the magneto-actuated electrochemical immunosensor for the detection of exosomes spiked on PBS (10 mmol L^−1^) and on human serum (exosome-depleted), and performed by IMS on antiCD81-MPs and the detection of cancer-related biomarkers (CD24 and CD340 for Figure 10, panels A and B, respectively).

The limit of detection (LOD) for each plot were estimated by fitting the raw data using a nonlinear regression (four parameters logistic equation), by processing the negative control samples (n = 10) and obtaining the mean value for each plot. The cutoff value was then determined in all cases with a one-tailed t-test at a 95% confidence level. These values were interpolated in each plot to obtain the LODs.

The LOD for the detection approach using antiCD24 was found to be 1.94 × 10^5^ and 1.73 × 10^5^ exosomes per µL^−1^ for exosomes spiked in human serum and phosphate buffer, respectively. The LOD for the detection approach using antiCD340 was found to be 1.02 × 10^6^ and 5.64 × 10^5^ exosomes per µL^−1^ for exosomes spiked in human serum and PBS, respectively.

Finally, the immunosensor for CD63 recognition, a LOD of 1.24 × 10^5^ and as low as 2.34 × 10^4^ exosomes per µL^−1^ was obtained with antiCD63-HRP (Figure 10, panel C) in non-diluted human serum and a phosphate buffer. Although from the results shown in Figure 10, there is a matrix effect in the serum, this approach demonstrated a good performance of the electrochemical immunosensor towards cancer-related exosomes isolated by IMS in non-diluted human serum samples, without any further pretreatment (such as ultracentrifugation).

## 4. Conclusions

The most important feature that should be considered to simplify the analytical procedure for the detection of exosomes at low concentration levels involves novel solid-phase separation methods to avoid ultracentrifugation. In this paper, we demonstrated that particle-based magnetic enrichment simplifies exosomes isolation and can be easily coupled with emerging technologies as is the case with electrochemical immunosensing. However, the interferences of free receptors present in the serum, including the tetraspanins CD9 and CD63, can prevent or interfere with the immunomagnetic separation based on these receptors. Interestingly, the immunomagnetic separation of the exosomes based on CD81 is not affected by the serum (even if it is undiluted), which can be easily detected by an electrochemical immunosensor using cancer related biomarkers, such as CD24 and CD340.

Moreover, the exosome electrochemical immunosensor shows an outstanding LOD as low as 10^4^ exosomes per μL^−1^ directly in undiluted human serum when the detection is based on CD63, this LOD being compatible with the levels of exosomes present in serum and plasma and, importantly, without any previous isolation step using ultracentrifugation. The electrochemical immunosensor, coupled with immunomagnetic separation, offers an exciting alternative, especially in resource-scarce settings that can be handled by unskilled personnel at the community care level.

## Figures and Tables

**Table 1:** Summary of the antibodies used in this work.

Antibody.	Target	Clonality	Conjugate	Host	Reference	Commercial Source	Use/s
antiCD24	CD24	monoclonal	no	mouse	ab76514	Abcam	1, 2, 3
antiCD54	CD54	monoclonal	no	mouse	ab2213	Abcam	1, 2, 3
antiCD326	CD326	monoclonal	no	mouse	ab7504	Abcam	1, 2, 3
antiCD340	CD340	monoclonal	no	mouse	Ab30	Abcam	1, 2, 3
antiCD9	CD9	monoclonal	no	mouse	10626D	Thermo Fisher	1, 2, 3
antiCD63	CD63	monoclonal	no	mouse	10628D	Thermo Fisher	1, 2, 3
antiCD81	CD81	monoclonal	no	mouse	10630D	Thermo Fisher	1, 2, 3
antiCD44	CD81	monoclonal	no	mouse	BMS113	eBioscience	1, 2, 3
antiCD81	CD81	polyclonal	no	rabbit	HPA007234	Sigma-Aldrich	1, 2, 3
antimouse-HRP	mouse IgG H&L	polyclonal	HRP	rabbit	ab6728	Abcam	4
antimouse-Cy5	mouse IgG H&L	polyclonal	Cy5^®^	rabbit	ab97037	Abcam	5
antiCD63-HRP	CD63	monoclonal	HRP	mouse	NBP2-42225H-100	BioNova	6

Uses: 1 Immunomagnetic separation when attached to tosyl-activated magnetic particles. 2 Indirect labeling in electrochemical immunosensing, as a primary antibody. 3 Indirect labeling in confocal microscopy and flow cytometry, as a primary antibody. 4 Indirect labeling in magneto-actuated enzyme-linked immunosorbent assay (magneto-ELISA) and electrochemical immunosensing, as a secondary antibody. 5 Indirect labeling in confocal microscopy and flow cytometry, as a secondary antibody. 6 Direct labeling in magneto-ELISA and electrochemical immunosensing, as a secondary antibody.

**Table 2:** Description of the biomarkers selected in this work.

Biomarker	Length/Mass *	Description *	Function *	Most Prominent Carcinomas Reported in 2018 **
CD9	141–228 aa/15.9–25.4 kDa	Cell-surface proteins member of the transmembrane 4 superfamily, also known as the tetraspanin family. Tetraspanins are extensively and variably glycosylated.	Involved in cell adhesion, migration and motility and also platelet activation and aggregation.	Breast [24]/Prostate [25]/Lung [26]/Colorectum [27]/Stomach [28]/Liver [29]
CD63	133–238 aa/14.3–25.6 kDa			Breast [30]/Prostate [31]/Lung [32]/Colorectum [33]/Stomach [34]/Liver [35]
CD81	51–274 aa/5.6–29.8 kDa			Breast [36]/Prostate [37]/Lung [38]/Colorectum [39]/Stomach [40]/Liver [41]
CD24	37–129 aa/4.1–13 kDa	Cell-surface small protein that is heavily and distinctly glycosylated. It is a sialoglycoprotein expressed in B-cell development and B-cell neoplasia and a large variety of malignant tumors.	Involved in the regulation of cell binding capacity, proliferation, maturation and tumor metastasis.	Breast [42]/Prostate [43]/Lung [44]/Colorectum [45]/Stomach [46]/Liver [47]
CD44	78–742 aa/8.8–81.5 kDa	Cell-surface transmembrane glycoprotein, receptor for hyaluronic acid (HA) and other ligands, such as osteopontin, collagens and matrix metalloproteinases (MMPs). Frequently called HCAM (homing cell adhesion molecule)	Involved in in cell activation, proliferation, differentiation, recirculation and homing, hematopoiesis and tumor metastasis.	Breast [48]/Prostate [49]/Lung [50]/Colorectum [51]/Stomach [52]/Liver [53]
CD54 (ICAM-1, Intercellular Adhesion Molecule 1)	180–532 aa/19.4–57.8 kDa	Cell-surface transmembrane glycoprotein of the immunoglobulin superfamily. Typically expressed on endothelial cells and also found in epithelial cells and cells of the immune system. Frequently called ICAM-1 (intercellular adhesion molecule 1).	Involved in leukocyte endothelial transmigration, cell signaling, adhesion, polarity, cell-cell interaction, tissue stability and tumor metastasis.	Breast [54]/Prostate [55]/Lung [56]/Colorectum [57]/Stomach [58]/Liver [59]
CD326 (Epithelial cell adhesion molecule (EpCAM)	199–342 aa/21–37.9 kDa	Cell-surface transmembrane glycoprotein that mediates cell adhesion in epithelia. Plays a role in tumorigenesis and metastasis of carcinomas. It is also frequently called EpCAM (epithelial cell adhesion molecule).	Involved in cell signaling, migration, proliferation, differentiation and tumor metastasis.	Breast [60]/Prostate [61]/Lung [62]/Colorectum [63]/Stomach [64]/Liver [65]
CD340 (HER2/erbB2, tyrosine kinase cell surface receptor HER2, oncogene ERBB2)	102–1255 aa/11.5–137.9 kDa	Cell-surface transmembrane glycoprotein that is a receptor for members of the epidermal growth factor (EGF) family. Plays a role in tumorigenesis and metastasis of carcinomas. Frequently called HER2 (human epidermal growth factor receptor 2).	Involved in the binding with the ligand-bound epidermal growth factor (EGF) receptor family members, enhancing kinase-mediated activation of downstream signaling pathways and tumor metastasis.	Breast [66]/Prostate [67]/Lung [68]/Stomach [69]/Liver [70]

* Data were taken from The Human Protein Atlas; https://www.proteinatlas.org/. ** Most prominent carcinomas in 2018 were taken from the Global Cancer Observatory: https://gco.iarc.fr/.

## References

[1] Bray F., Ferlay J., Soerjomataram I., Siegel R.L., Torre L.A., Jemal A. (2018). Global cancer statistics 2018: GLOBOCAN estimates of incidence and mortality worldwide for 36 cancers in 185 countries. Cancer J. Clin..

[2] Johnstone R.M., Adam M., Hammond J.R., Orr L., Turbide C. (1987). Vesicle formation during reticulocyte maturation. Association of plasma membrane activities with released vesicles (exosomes). J. Boil. Chem..

[3] Halvaei S., Daryani S., Eslami-S Z., Samadi T., Jafarbeik-Iravani N., Bakhshayesh T.O., Majidzadeh-A K., Esmaeili R. (2018). Exosomes in Cancer Liquid Biopsy: A Focus on Breast Cancer. Mol. Ther.-Nucleic Acids.

[4] Trams E.G., Lauter C.J., Salem J.N., Heine U. (1981). Exfoliation of membrane ecto-enzymes in the form of micro-vesicles. Biochim. Biophys. Acta.

[5] Qi H., Liu C., Long L., Ren Y., Zhang S., Chang X., Qian X., Jia H., Zhao J., Sun J. (2016). Blood Exosomes Endowed with Magnetic and Targeting Properties for Cancer Therapy. ACS Nano.

[6] Sun Y., Xia Z., Shang Z., Sun K., Niu X., Qian L., Fan L.-Y., Cao C.-X., Xiao H. (2016). Facile preparation of salivary extracellular vesicles for cancer proteomics. Sci. Rep..

[7] Fenner A. (2016). Biomarkers: Urinary exosome biomarkers of radiation exposure. Nat. Rev. Urol..

[8] Zhang H.-G., Grizzle W.E. (2014). Exosomes. Am. J. Pathol..

[9] Patel G.K., Khan M.A., Zubair H., Srivastava S.K., Khushman M., Singh S., Singh A.P. (2019). Comparative analysis of exosome isolation methods using culture supernatant for optimum yield, purity and downstream applications. Sci. Rep..

[10] Théry C., Amigorena S., Raposo G., Clayton A. (2016). Isolation and Characterization of Exosomes from Cell Culture Supernatants and Biological Fluids. Curr. Protoc. Cell Biol..

[11] Rembaum A., Yen R., Kempner D., Ugelstad J. (1982). Cell labeling and magnetic separation by means of immunoreagents based on polyacrolein microspheres. J. Immunol. Methods.

[12] Reddy L.H., Arias J.L., Nicolas J., Couvreur P., Patrick C. (2012). Magnetic Nanoparticles: Design and Characterization, Toxicity and Biocompatibility, Pharmaceutical and Biomedical Applications. Chem. Rev..

[13] Lermo A., Zacco E., Barak J., Delwiche M., Campoy S., Barbe J., Alegret S., Pividori M. (2008). Towards Q-PCR of pathogenic bacteria with improved electrochemical double-tagged genosensing detection. Biosens. Bioelectron..

[14] Brandao D., Liebana S., Pividori M.I. (2015). Multiplexed detection of foodborne pathogens based on magnetic particles. New Biotechnol..

[15] Carinelli S., Martí M., Alegret S., Pividori M.I. (2015). Biomarker detection of global infectious diseases based on magnetic particles. New Biotechnol..

[16] Carinelli S., Xufré C., Marti M., Pividori M.I. (2018). Interferon gamma transcript detection on T cells based on magnetic actuation and multiplex double-tagging electrochemical genosensing. Biosens. Bioelectron..

[17] Moura S.L., García Martín C., Martí M., Pividori M.I. (2020). Multiplex detection and characterization of breast cancer exosomes by magneto-actuated immunoassay. Talanta.

[18] Weissleder R., Pittet M.J. (2008). Imaging in the era of molecular oncology. Nature.

[19] Allard W.J., Matera J., Miller M.C., Repollet M., Connelly M.C., Rao C., Tibbe A.G., Uhr J.W., Terstappen L.W. (2004). Tumor cells circulate in the peripheral blood of all major carcinomas but not in healthy subjects or patients with nonmalignant diseases. Clin. Cancer Res..

[20] De Wit S., Manicone M., Rossi E., Lampignano R., Yang L., Zill B., Rengel-Puertas A., Ouhlen M., Crespo M., Berghuis A.M.S. (2018). EpCAMhigh and EpCAMlow circulating tumor cells in metastatic prostate and breast cancer patients. Oncotarget.

[21] Ross A.A., Cooper B.W., Lazarus H.M., Mackay W., Moss T.J., Ciobanu N., Tallman M.S., Kennedy M.J., Davidson N.E., Sweet D. (1993). Detection and viability of tumor cells in peripheral blood stem cell collections from breast cancer patients using immunocytochemical and clonogenic assay techniques. Blood.

[22] Wang L., Balasubramanian P., Chen A.P., Kummar S., Evrard Y.A., Kinders R.J. (2016). Promise and limits of the CellSearch platform for evaluating pharmacodynamics in circulating tumor cells. Semin. Oncol..

[23] Chiu Y.-J., Cai W., Shih Y.-R.V., Lian I., Lo Y.-H. (2016). A Single-Cell Assay for Time Lapse Studies of Exosome Secretion and Cell Behaviors. Small.

[24] Rappa G., Green T.M., Karbanová J., Corbeil D., Lorico A. (2015). Tetraspanin CD9 determines invasiveness and tumorigenicity of human breast cancer cells. Oncotarget.

[25] Zvereff V., Wang J.-C., Shun K., Lacoste J., Chevrette M. (2007). Colocalisation of CD9 and mortalin in CD9-induced mitotic catastrophe in human prostate cancer cells. Br. J. Cancer.

[26] Kohmo S., Kijima T., Otani Y., Mori M., Minami T., Takahashi R., Nagatomo I., Takeda Y., Kida H., Goya S. (2010). Cell Surface Tetraspanin CD9 Mediates Chemoresistance in Small Cell Lung Cancer. Cancer Res..

[27] Kim K.-J., Kwon H.J., Kim M.C., Bae Y.K. (2016). CD9 Expression in Colorectal Carcinomas and Its Prognostic Significance. J. Pathol. Transl. Med..

[28] Murayama Y., Oritani K., Tsutsui S. (2015). Novel CD9-targeted therapies in gastric cancer. World J. Gastroenterol..

[29] Zheng R., Yano S., Zhang H., Nakataki E., Tachibana I., Kawase I., Hayashi S., Sone S. (2005). CD9 overexpression suppressed the liver metastasis and malignant ascites via inhibition of proliferation and motility of small-cell lung cancer cells in NK cell-depleted SCID mice. Oncol. Res..

[30] Tominaga N., Hagiwara K., Kosaka N., Honma K., Nakagama H., Ochiya T. (2014). RPN2-mediated glycosylation of tetraspanin CD63 regulates breast cancer cell malignancy. Mol. Cancer.

[31] Gong Y., Scott E., Lu R., Xu Y., Oh W.K., Yu Q. (2013). TIMP-1 Promotes Accumulation of Cancer Associated Fibroblasts and Cancer Progression. PLoS ONE.

[32] Kwon M.S., Shin S.-H., Yim S.-H., Lee K.Y., Kang H.-M., Kim T.-M., Chung Y.-J. (2007). CD63 as a biomarker for predicting the clinical outcomes in adenocarcinoma of lung. Lung Cancer.

[33] Wang X., Ding X., Nan L., Wang Y., Wang J., Yan Z., Zhang W., Sun J., Zhu W., Ni B. (2015). Investigation of the roles of exosomes in colorectal cancer liver metastasis. Oncol. Rep..

[34] Miki Y., Yashiro M., Okuno T., Kuroda K., Togano S., Hirakawa K., Ohira M. (2018). Clinico-pathological significance of exosome marker CD63 expression on cancer cells and stromal cells in gastric cancer. PLoS ONE.

[35] Cho Y.-E., Im E.-J., Moon P.-G., Mezey E., Song B.-J., Baek M.-C. (2017). Increased Liver-Specific Proteins in Circulating Extracellular Vesicles as Potential Biomarkers for Drug- and Alcohol-Induced Liver Injury. PLoS ONE.

[36] Zhang N., Zuo L., Zheng H., Li G., Hu X. (2018). Increased Expression of CD81 in Breast Cancer Tissue is Associated with Reduced Patient Prognosis and Increased Cell Migration and Proliferation in MDA-MB-231 and MDA-MB-435S Human Breast Cancer Cell Lines In Vitro. Med Sci. Monit..

[37] Logozzi M., Angelini D.F., Iessi E., Mizzoni D., Di Raimo R., Federici C., Lugini L., Borsellino G., Gentilucci A., Pierella F. (2017). Increased PSA expression on prostate cancer exosomes in in vitro condition and in cancer patients. Cancer Lett..

[38] Guilmain W., Colin S., Legrand E., Vannier J.P., Steverlynck C., Bongaerts M., Vasse M., Al-Mahmood S. (2011). CD9P-1 expression correlates with the metastatic status of lung cancer, and a truncated form of CD9P-1, GS-168AT2, inhibits in vivo tumour growth. Br. J. Cancer.

[39] Chiba M., Kimura M., Asari S. (2012). Exosomes secreted from human colorectal cancer cell lines contain mRNAs, microRNAs and natural antisense RNAs, that can transfer into the human hepatoma HepG2 and lung cancer A549 cell lines. Oncol. Rep..

[40] Yoo T.-H., Ryu B.-K., Lee M.-G., Chi S.-G. (2013). CD81 Is a Candidate Tumor Suppressor Gene in Human Gastric Cancer. Cell. Oncol..

[41] Bruening J., Lasswitz L., Banse P., Kahl S., Marinach C., Vondran F.W., Kaderali L., Silvie O., Pietschmann T., Meissner F. (2018). Hepatitis C virus enters liver cells using the CD81 receptor complex proteins calpain-5 and CBLB. PLOS Pathog..

[42] Fang X., Zheng P., Tang J., Liu Y. (2010). CD24: From A to Z. Cell. Mol. Immunol..

[43] Zhang Y., Li B., Zhang X., Sonpavde G.P., Jiao K., Zhang A., Zhang G., Sun M., Chu C., Li F. (2017). CD24 Is a Genetic Modifier for Risk and Progression of Prostate Cancer. Mol. Carcinog..

[44] Kristiansen G., Schlüns K., Yongwei Y., Denkert C., Dietel M., Petersen I. (2003). CD24 is an independent prognostic marker of survival in nonsmall cell lung cancer patients. Br. J. Cancer.

[45] Ke J., Wu X., Wu X., He X., Lian L., Zou Y., He X., Wang H., Luo Y., Wang L. (2012). A Subpopulation of CD24+ Cells in Colon Cancer Cell Lines Possess Stem Cell Characteristics. Neoplasma.

[46] Wang Y.-C., Wang J.-L., Kong X., Sun T.-T., Chen H.-Y., Hong J., Fang J.-Y. (2014). CD24 Mediates Gastric Carcinogenesis and Promotes Gastric Cancer Progression via STAT3 Activation. Apoptosis.

[47] Lee T.K.W., Castilho A., Cheung V.C.H., Tang K.H., Ma S., Ng I.O.L. (2011). CD24(+) Liver Tumor-Initiating Cells Drive Self-Renewal and Tumor Initiation through STAT3-Mediated NANOG Regulation. Cell Stem Cell.

[48] Louderbough J.M.V., Schroeder J.A. (2011). Understanding the Dual Nature of CD44 in Breast Cancer Progression. Mol. Cancer Res..

[49] Iczkowski K.A. (2010). Cell adhesion molecule CD44: Its functional roles in prostate cancer. Am. J. Transl. Res..

[50] Hu B., Ma Y., Yang Y., Zhang L., Han H., Chen J. (2018). CD44 promotes cell proliferation in non-small cell lung cancer. Oncol. Lett..

[51] Shiozawa M., Guan H.-B., Tanaka K., Endo I., Akaike M., Zheng Y.-W., Taniguchi H., Watanabe K., Miyata H., Ozawa M. (2014). Prognostic significance of CD44 variant 2 upregulation in colorectal cancer. Br. J. Cancer.

[52] Heo D., Huh Y.-M., Yang J., Yang S.-H., Suh J.-S., Lee H., Son H., Haam S. (2015). Molecular Imaging of CD44-Overexpressing Gastric Cancer in Mice Using T2 MR Imaging. J. Nanosci. Nanotechnol..

[53] Dhar D., Antonucci L., Nakagawa H., Kim J.Y., Glitzner E., Caruso S., Shalapour S., Yang L., Valasek M.A., Lee S. (2018). Liver Cancer Initiation Requires p53 Inhibition by CD44-Enhanced Growth Factor Signaling. Cancer Cell.

[54] Guo P., Huang J., Wang L., Jia D., Yang J., Dillon D.A., Zurakowski D., Mao H., Moses M.A., Auguste D.T. (2014). ICAM-1 as a molecular target for triple negative breast cancer. Proc. Natl. Acad. Sci. USA.

[55] Li C., Liu S., Yan R., Han N., Wong K.-K., Li L. (2017). CD54-NOTCH1 axis controls tumor initiation and cancer stem cell functions in human prostate cancer. Theranostics.

[56] Gerbitz A., Ewing P., Olkiewicz K., Willmarth N.E., Williams D., Hildebrandt G., Wilke A., Liu C., Eissner G., Andreesen R. (2005). A Role for CD54 (Intercellular Adhesion Molecule-1) in Leukocyte Recruitment to the Lung During the Development of Experimental Idiopathic Pneumonia Syndrome. Transplantation.

[57] Fang C., Zhou Z., Hu J., Mo X., Li Y., Fan C., Huang Q., Yang L., Peng Z., Meng W. (2016). CD133+CD54+CD44+ Circulating Tumor Cells as a Biomarker of Treatment Selection and Liver Metastasis in Patients with Colorectal Cancer. Oncotarget.

[58] Yashiro M., Sunami T., Hirakawa K. (2005). CD54 Expression Is Predictive for Lymphatic Spread in Human Gastric Carcinoma. Dig. Dis. Sci..

[59] Liu S., Li N., Yu X., Xiao X., Cheng K., Hu J., Wang J., Zhang D., Cheng S., Liu S. (2013). Expression of Intercellular Adhesion Molecule 1 by Hepatocellular Carcinoma Stem Cells and Circulating Tumor Cells. Gastroenterology.

[60] Vang K.B., Jenkins S.V., Zharov V.P., Griffin R.J., Nima Z.A., Nedosekin D.A., Dings R.P.M., Kannarpady G., Biris A.S. (2017). Triple-negative breast cancer targeting and killing by EpCAM-directed, plasmonically active nanodrug systems. Precis. Oncol..

[61] Ni J., Cozzi P., Beretov J., Duan W., Bucci J., Graham P., Li Y. (2018). Epithelial cell adhesion molecule (EpCAM) is involved in prostate cancer chemotherapy/radiotherapy response in vivo. BMC Cancer.

[62] Hasegawa K., Sato A., Tanimura K., Uemasu K., Hamakawa Y., Fuseya Y., Sato S., Muro S., Hirai T. (2017). Fraction of MHCII and EpCAM expression characterizes distal lung epithelial cells for alveolar type 2 cell isolation. Respir. Res..

[63] Nicolazzo C., Raimondi C., Francescangeli F., Ceccarelli S., Trenta P., Magri V., Marchese C., Zeuner A., Gradilone A., Gazzaniga P. (2017). EpCAM-Expressing Circulating Tumor Cells in Colorectal Cancer. Int. J. Boil. Markers.

[64] Dai M., Yuan F., Fu C., Shen G., Hu S., Shen G. (2017). Relationship between Epithelial Cell Adhesion Molecule (EpCAM) Overexpression and Gastric Cancer Patients: A Systematic Review and Meta-Analysis. PLoS ONE.

[65] Matsumoto T., Takai A., Eso Y., Kinoshita K., Manabe T., Seno H., Chiba T., Marusawa H. (2017). Proliferating EpCAM-Positive Ductal Cells in the Inflamed Liver Give Rise to Hepatocellular Carcinoma. Cancer Res..

[66] Levva S., Kotoula V., Kostopoulos I., Manousou K., Papadimitriou C., Papadopoulou K., Lakis S., Koukoulias K., Karavasilis V., Pentheroudakis G. (2017). Prognostic Evaluation of Epidermal Growth Factor Receptor (EGFR) Genotype and Phenotype Parameters in Triple-negative Breast Cancers. Cancer Genom.-Proteom..

[67] Day K.C., Hiles G.L., Kozminsky M., Dawsey S.J., Paul A., Broses L.J., Shah R., Kunja L.P., Hall C., Palanisamy N. (2017). HER2 and EGFR Overexpression Support Metastatic Progression of Prostate Cancer to Bone. Cancer Res..

[68] Bethune G., Bethune E., Ridgway N., Xu Z. (2010). Epidermal growth factor receptor (EGFR) in lung cancer: An overview and update. J. Thorac. Dis..

[69] Galizia G., Lieto E., Orditura M., Castellano P., La Mura A., Imperatore V., Pinto M., Zamboli A., De Vita F., Ferraraccio F. (2007). Epidermal Growth Factor Receptor (EGFR) Expression is Associated With a Worse Prognosis in Gastric Cancer Patients Undergoing Curative Surgery. World J. Surg..

[70] Komposch K., Sibilia M. (2016). EGFR Signaling in Liver Diseases. Int. J. Mol. Sci..

[71] Xu J., Mahajan K., Xue W., Winter J.O., Zborowski M., Chalmers J.J. (2012). Simultaneous, single particle, magnetization and size measurements of micron sized, magnetic particles. J. Magn. Magn. Mater..

[72] Hoogenboom R., Fijten M.W.M., Kickelbick G., Schubert U.S. (2010). Synthesis and crystal structures of multifunctional tosylates as basis for star-shaped poly(2-ethyl-2-oxazoline)s. Beilstein J. Org. Chem..

[73] Filipe V., Hawe A., Jiskoot W. (2010). Critical Evaluation of Nanoparticle Tracking Analysis (NTA) by NanoSight for the Measurement of Nanoparticles and Protein Aggregates. Pharm. Res..

[74] Li Q., Tofaris G.K., Davis J.J. (2017). Concentration-Normalized Electroanalytical Assaying of Exosomal Markers. Anal. Chem..

[75] Bamgbelu A., Wang J., Leszczynski J. (2010). TDDFT Study of the Optical Properties of Cy5 and Its Derivatives. J. Phys. Chem. A.

[76] Chosewood L.C., Wilson D.E. (2009). Biosafety in Microbiological and Biomedical Laboratories.

[77] Agrawal A., Sathe T., Nie S. (2007). Single-Bead Immunoassays Using Magnetic Microparticles and Spectral-Shifting Quantum Dots. J. Agric. Food Chem..

[78] Hemler M.E. (2013). Tetraspanin proteins promote multiple cancer stages. Nat. Rev. Cancer.

[79] Chow A., Zhou W., Liu L., Fong M.Y., Champer J., Van Haute D., Chin A.R., Ren X., Gugiu B.G., Meng Z. (2015). Macrophage immunomodulation by breast cancer-derived exosomes requires Toll-like receptor 2-mediated activation of NF-κB. Sci. Rep..

[80] Abu-Seer E. (2017). The Reliability of Plasma Exosome Concentrations in Healthy Male Individuals. J. Heal. Sci..

[81] Fernando M.R., Jiang C., Krzyzanowski G.D., Ryan W.L. (2017). New evidence that a large proportion of human blood plasma cell-free DNA is localized in exosomes. PLoS ONE.

[82] Soares Martins T., Catita J., Martins Rosa I., da Cruz e Silva O.A.B., Henriques A.G. (2018). Exosome isolation from distinct biofluids using precipitation and column-based approaches. PLoS ONE.

[83] Huang X., Yuan T., Tschannen M., Sun Z., Jacob H., Du M., Liang M., Dittmar R.L., Liu Y., Liang M. (2013). Characterization of human plasma-derived exosomal RNAs by deep sequencing. BMC Genom..

